# Cytomegalovirus Retinitis Following Daratumumab for Multiple Myeloma: A Case Report and Literature Review

**DOI:** 10.1155/carm/8861458

**Published:** 2025-09-04

**Authors:** Asuka Kono, Kana Bando, Atsushi Takahata, Shigeo Toyota

**Affiliations:** Department of Hematology, Yokosuka Kyosai Byoin, Yokosuka, Kanagawa, Japan

**Keywords:** cytomegalovirus infection, cytomegalovirus retinitis, daratumumab, multiple myeloma

## Abstract

**Introduction:** Cytomegalovirus (CMV) retinitis typically occurs in patients with acquired immunodeficiency syndrome. It may also manifest in patients with hematological diseases, mostly after allogeneic hematopoietic cell transplantation. However, its incidence in multiple myeloma remains exceedingly rare, with only 15 reported cases in the literature.

**Case Report:** A 71-year-old man diagnosed with multiple myeloma achieved complete response following treatment with daratumumab, lenalidomide, and dexamethasone. After 2 years of therapy, he developed CMV retinitis. Systemic antiviral treatment led to improved visual acuity, and antimyeloma treatment was successfully resumed with reduced intensity of chemotherapy.

**Conclusion:** CMV reactivation is increasingly being reported with the development of new treatment strategies for multiple myeloma and is considered a significant drug-related clinical complication. We reviewed previous reports and discussed the incidence and optimal management of CMV reactivation in this patient population.

## 1. Introduction

Cytomegalovirus (CMV) retinitis is a vision-threatening ocular disease commonly observed in patients with acquired immune deficiency syndrome (AIDS) with profound CD4 lymphocytopenia. In hematological disorders, CMV retinitis is predominantly associated with allogeneic hematopoietic stem cell transplantation (allo-HSCT) [1]. CMV disease affecting organ dysfunction is rarely observed in patients with hematological malignancies in the absence of allo-HSCT [2]. Current guidelines do not recommend routine monitoring or prophylaxis for CMV in multiple myeloma due to limited data [3, 4]. However, emerging evidence suggests a relatively high incidence of CMV infection with the development of new treatments for multiple myeloma, especially anti-CD38 monoclonal antibodies (mAbs) [5–10].

Herein, we describe a rare case of CMV retinitis following daratumumab treatment for multiple myeloma and provide a comprehensive review of previous reports on CMV reactivation in multiple myeloma. This case and literature review provide new insights into the optimal management of CMV in multiple myeloma.

## 2. Case Report

A 71-year-old male with a medical history of chronic kidney disease, prostate cancer, and hypertension was found to have anemia and peripheral plasmacytosis during surveillance for prostate cancer in August 2020. Laboratory findings revealed anemia (hemoglobin: 7.7 g/dL), leukocytosis (white blood cells count: 15,000/mm^3^) with 31% circulating plasma cells, and marked hypogammaglobulinemia. Serum immunofixation demonstrated monoclonal BJP-lambda, and the level of free light chain (FLC)-lambda was markedly elevated at 1270 mg/dL. Bone marrow aspirate revealed 80% plasma cell infiltration. Chromosomal karyotyping revealed a normal karyotype, and fluorescence in situ hybridization (FISH) revealed no high-risk chromosomal aberrations such as del(17p13.1), t(4; 14), or t(14; 16). Computed tomography identified multiple osteolytic lesions. The patient was diagnosed with multiple myeloma (FLC-lambda type, plasma cell leukemia type, and R-ISS II). Given the history of docetaxel-induced neuropathy, bortezomib was avoided, and DRd (daratumumab 1800 mg, lenalidomide 10 mg, and dexamethasone 40 mg weekly) was initiated. DRd chemotherapy rapidly reduced FLC levels and achieved a stringent complete response, which was maintained for 20 months without infectious complications. Acyclovir 200 mg/day was administered as antiviral prophylaxis. We did not administer prophylactic immunoglobulin supplementation for hypogammaglobulinemia (IgG, approximately 200 mg/dL), considering no history of infectious complications.

The patient noted blurred vision in his left eye and visited our ophthalmologist in February 2023 during the 21st cycle of DRd. Initial management for presumed uveitis with local steroids and levofloxacin was ineffective, and his visual disturbance and fundus findings worsened. Therefore, the patient was transferred to a specialized institution for further investigation. Ophthalmic examination revealed a left best-corrected visual acuity (BCVA) of 6/7.5 (right 6/6). Fundus examination of the left eye revealed vitreous opacity, retinal infiltrates, and occlusive vasculitis. The differential diagnoses included infections such as CMV retinitis, tuberculosis, toxoplasma, and neoplastic infiltration. Polymerase chain reaction (PCR) of aqueous humor confirmed CMV DNA (10,000 copies/mL), leading to the diagnosis of CMV retinitis.

**Figure 1:**
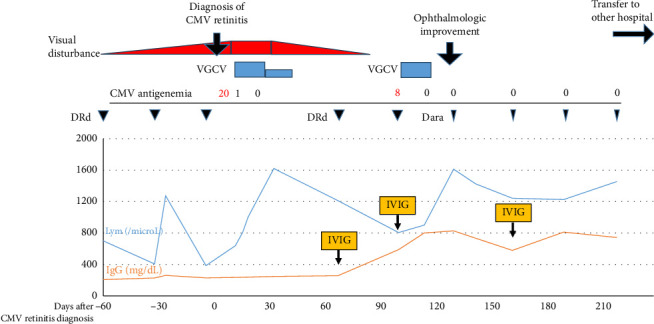
Clinical course of the patient. CMV, cytomegalovirus; Dara, daratumumab; DRd, daratumumab, lenalidomide, and dexamethasone; IVIG, intravenous immunoglobulin; Lym, lymphocyte; VGCV, valganciclovir.

The patient had lymphocytopenia (300–1000/mm^3^) with a slightly low CD4 count (426/mm^3^) and IgG levels of 220 mg/dL. Serological tests for human immunodeficiency virus, human T-cell leukemia virus type-1, and toxoplasma were negative. The CMV pp65 antigenemia assay was positive (20/50,000 cells). After the diagnosis of CMV retinitis, chemotherapy was interrupted, and oral valganciclovir (450 mg/day adjusted for renal impairment) was initiated and continued for 4 weeks, including a maintenance phase. Although short-term follow-up showed no remarkable change in visual acuity, CMV-DNA of the anterior chamber became undetectable after 34 days of treatment, and fundoscopic examination 4 months later showed resolution of vitreous haze and improved vision. Intravenous immunoglobulin was initiated to maintain IgG above 400 mg/dL. Two months later, DRd was resumed with reduced dexamethasone (20 mg monthly) and CMV monitoring. CMV antigenemia on Day 28 turned positive but resolved with short-term VGCV without exacerbation of visual disturbance or ophthalmic findings. Daratumumab monotherapy was subsequently resumed without recurrence of CMV retinitis for 8 months. Valganciclovir prophylaxis was discontinued due to insufficient evidence. After reduction to daratumumab monotherapy, lymphocytopenia improved, and IgG levels were maintained above 400 mg/dL with monthly immunoglobulin supplementation. The patient was later transferred to another hospital. Their clinical course after the diagnosis of CMV retinitis and immunologic parameters are illustrated in Figure 1.

## 3. Discussion

Case reports of CMV disease related to multiple myeloma are limited only to 15 for retinitis [6, 11–20], 5 colitis, 1 pneumonia, and 1 hepatitis following a literature review of PubMed and MEDLINE databases [6, 19, 21, 22]. These 15 cases and our case of CMV retinitis are summarized in Table 1. Preceding chemotherapy included mAbs, immunomodulatory drugs, autologous HSCT, and chimeric antigen receptor T-cell therapy (CAR-T). Many patients had hypogammaglobulinemia and CD4 lymphocytopenia [12, 14, 17–20]. While many cases were heavily treated, three cases developed CMV retinitis during first-line treatment [15, 17, 18], similar to our case. All cases received systemic antiviral treatment, and some also received intravitreal therapy. The optimal duration of antiviral treatment for CMV retinitis remains undefined. Among the four patients who resumed chemotherapy, one experienced CMV retinitis relapse [18] and another had a prolonged duration of CMV antigenemia requiring antiviral treatment for 154 days [19]. Teheh et al. [18] reported a case with prolonged CD4 lymphocytopenia that could not be initiated on chemotherapy, resulting in death from the progression of multiple myeloma. Monitoring and prophylaxis for CMV, as well as the use of prophylactic immunoglobulin after CMV disease, were not described in these case reports.

In our case, the CD4 count at CMV diagnosis was maintained above the AIDS-related risk threshold of 50/mm^3^ [23]. We postulated that prolonged hypogammaglobulinemia and ongoing chemotherapy (particularly daratumumab and high-dose steroids) predisposed the patient to CMV retinitis. Currently, there is no optimal chemotherapeutic strategy for multiple myeloma with CMV infection. We decided to continue reduced chemotherapy with careful monitoring for CMV infection, considering the generally poor prognosis of plasma cell leukemia, especially in relapsed cases.

CMV reactivation in multiple myeloma is usually associated with allo-HSCT and manifest as viremia rather than end-organ disease [24, 25]. However, several retrospective studies indicate a substantial incidence of CMV reactivation in nontransplant settings, especially following exposure to mAbs, CAR-T, and bispecific antibodies (bsAbs) (Table 2) [5–11, 24–29]. Reported incidence rates of CMV infection range from 17% to 73%, reflecting the difference in patient background (previous treatment and CMV surveillance protocols). Li et al. [8] suggested that an early decrease in CD56+ NK cells and CD8+ T cells contributes to CMV reactivation within a few months of daratumumab initiation, which is consistent with other studies [6–9]. While lymphocytopenia, hypogammaglobulinemia, and prior allo-HSCT are reported risk factors, statistical validation remains limited due to small sample sizes. Although most reported cases did not progress to CMV disease, antiviral therapy was employed in many studies (Table 2). Preemptive strategies were largely following the guidelines of each institution or the discretion of the attending physician. CMV infection can necessitate chemotherapy interruption, increasing the risk of multiple myeloma progression. Matsunaga et al. [6] reported that 7 patients discontinued chemotherapy and 15 required dose reduction or treatment delay among 29 patients with CMV infection.

The treatment of multiple myeloma has undergone a paradigm shift with the increasing use of immunotherapies, such as CAR-T and bsAbs. In general, CAR-T and bsAbs are at increased risk of infection due to various factors, including multiple previous lines of chemotherapy, and prolonged cytopenia and hypogammaglobulinemia. CMV reactivation rates following BCMA-CAR-T therapy have been reported to range from 10% to 33%, even with immunoglobulin prophylaxis [30, 31]. Kikuchi et al. [11] reported a 38.9% (20/53) incidence of CMV reactivation during weekly monitoring, and 8 patients required antiviral therapy. Regarding bsAbs, in a cross-sectional analysis of its use for multiple myeloma, CMV infection was reported in 8% of 1185 patients [32]. An interim analysis of a prospective study [27] reported that 64% of 22 patients exposed to mAbs or bsAbs had detectable CMV-DNA during regular monitoring, and another study [26] reported a similarly high rate of CMV reactivation (52.2% of 23 patients), implicating the potential high risk of CMV reactivation following bsAbs.

In the current guidelines [3, 4], monitoring or prophylaxis for CMV is not recommended for patients with myeloma. Given the considerable frequency and clinical significance of CMV infection reported in previous studies, routine screening and preemptive therapy for CMV reactivation may be warranted in high-risk patients. Further large prospective studies are required to determine the risk factors and optimize monitoring and prophylactic strategies.

Herein, we describe a case of CMV disease following the treatment of multiple myeloma. This case and previous reports highlight the relatively high incidence and clinical significance of CMV reactivation in multiple myeloma.

## 4. Conclusion

In conclusion, we report a rare case of CMV retinitis following daratumumab therapy in a patient with multiple myeloma. This case, alongside a review of the literature, underscores the clinical relevance and growing incidence of CMV reactivation in this population. These findings support the need for heightened clinical vigilance and consideration of tailored monitoring and management protocols in high-risk patients.

## Figures and Tables

**Table 1:** Case reports of CMV retinitis in multiple myeloma.

Reference	Age	Preceding chemo (regimen/line)	CMV detection	IgG(mg/dL) Lym(/μL)	Treatment	Reinitiation of chemotherapy	Outcome
Ware et al. [13]	63	Dara, mezigdomide, dex	AC, PB	ND	FCN, VGCV	ND	Improvement (1 month)

Kikuchi et al. [11]	57	Ide-cel	PB	ALC 290	FCN, IVT	No	Improvement
	65	Ide-cel	No	ALC 570	GCV, IVT	No	Ablepsia
	36	Ide-cel	PB	ALC 340	VGCV	No	Improvement
	70	Ide-cel	PB	ALC 2110	VGCV	No	Improvement

Matsunaga et al. [6]	73	DCd/3rd	PB	ND	GCV	ND	Improvement (2 months)

Wang et al. [14]	58	Auto-HSCT, KPd	AC	CD4 87.5	VGCV 3 weeks, IVT, VGCV maintenance	ND	Improvement (5 months) Retinal hemorrhages, NVI

Zu et al. [12]	58	CAR-T, 5th	Vitreous, PB	IgG 210–249 ALC 3200	GCV 3 weeks, VGCV maintenance	ND	Improvement (1 month) Retinal detachment (5 months)

Cho et al. [20]	71	Dara, dex	Vitreous	CD4 450	GCV 3 weeks, VGCV 4 weeks, IVT	ND	Retinal hemorrhages (2 weeks)

Tanaka et al. [15]	63	DRd/1st	PB	ND	GCV 3 weeks maintenance	ND	No relapse (1 month)

Adams and Weng [16]	55	VRd, 8th	Vitreous	ND	VGCV, IVT	Yes (VGCV maintenance)	Improvement (2 months)

Lavi et al. [19]	36	DVd + Ven/7th	PB	IgG 170 ALC 280	VGCV	Yes (no detail)	CMV antigenemia Prolongation (154 days)

Lim et al. [17]	67	Len maintenance/1st	Vitreous	CD4 140	GCV 3 weeks, IVT VGCV maintenance	Yes (VGCV maintenance)	Improvement No relapse (9 months)

Teh et al. [18]	71	Rd, auto-HSCT/1st	Vitreous	CD4 50	GCV 3 weeks, IVT VGCV maintenance	Yes (VGCV maintenance)	No improvement CMV retinitis relapse
	69	2nd auto-HSCT/4th	Vitreous	CD4 100	GCV 4 months, IVT	No	Improvement (3 months) Death (PD)

Our case	71	DRd/1st	AC, PB	IgG 200–230 CD4 426	VGCV 2 wk No maintenance	Yes (dose reduction)	Improvement (3 months) No relapse (8 months)

*Note:* AC: anterior chamber of eye; Auto-HSCT: autologous hematopoietic stem cell transplantation; CAR-T: chimeric antigen receptor T cell therapy; CMV: cytomegalovirus; Dara: daratumumab; DCd: daratumumab/cyclophosphamide; DRd: daratumumab/lenalidomide/dexamethasone; DVd, daratumumab/bortezomib/dexamethasone; GCV, ganciclovir; IMiD: trial of CELMoD; KPd, carfilzomib/pomalidomide/dexamethasone; Len, lenalidomide; mo, month; Rd, lenalidomide/dexamethasone; Ven, venetoclax; VGCV, valganciclovir; VRd, bortezomib/lenalidomide/dexamethasone; wk, week.

Abbreviations: IVT, intravitreal therapy; ND, not described; PD, progressive disease.

**Table 2:** Retrospective studies of CMV reactivation in multiple myeloma.

Reference	Chemotherapy, total number in study	Regular CMV exam	CMV infection	CMV disease	Median amount of viremia	Antiviral treatment	Resume of chemotherapy
Kikuchi et al. [11]	Ide-cel, 53	Weekly	38.9% (20/53)	4 retinitis	ND	8/20	ND
Pei et al. [26]	Teclistamab, 23	ND	52.2% (12/23)	1 enterocolitis	DNA 2295 IU/mL (653-165000)	11/12	11/12
Baneman et al. [27]	CAR-T, 6 BsAb, 5 mAb, 11	Biweekly	64% (14/22)	No	DNA 34.5 IU/mL (34.5-14100)	1/14	ND
De Novellis et al. [5]	Dara, 51 Non-Dara 50	ND	Dara 33% (17/51) non-Dara 4% (2/51)	1 pneumonia	DNA 192 UI/mL (34.5-14100)	Dara 7/51 non-Dara 0/51	ND
Matsunaga et al. [6]	Dara, 154	Partial	19% (29/154)	1 hepatitis, 1 retinitis 1 gastroenteritis	Antigenemia 5 (2–27) cells/slide	25/29	7 discontinued
Kikuchi et al. [7]	Dara, 53	Partial	17% (9/53)	1 pneumonia	Antigenemia 4 (1–117) cells/slide	1/9	ND
Li et al. [8]	Dara, 53 Non-Dara, 78	Yes	Dara 17% (9/53) non-Dara 3% (2/78)	None	DNA 4360 cp/mL (1200–13700)	7/11	ND
Tabata et al. [9]	Dara, 15	Yes (1-2 times/wk)	73% (11/15)	None	Antigenemia 13 (2–104) cells/slide	8/11	ND
Nakagawa et al. [10]	Dara, 13	No	38% (5/13)	None	Antigenemia 12 (1–71)	5/5	1 postponed
Kikuchi et al. [28]	Elo, 85	ND	19% (16/85)	1 retinitis	ND	5/16	ND
Sharpley et al. [29]	Bor, 57	Yes (biweekly)	39% (12/31)	None	Median; ND DNA > 7500 cp/mL (5/12)	5/12	ND
Massoud et al. [25]	Auto-HSCT, 132	Yes (weekly)	22% (29/132)	3 enteritis	Median; ND DNA > 1000 cp/mL (11/29)	22/29	ND
Hasegawa et al. [24]	120	No	20% (24/120)	2 enteritis 1 gastritis	Antigenemia 2 (1–196) cells/slide	13/24	ND

*Note:* C: anterior chamber of eye; Auto-HSCT: autologous hematopoietic stem cell transplantation; CAR-T: chimeric antigen receptor T cell therapy; CMV: cytomegalovirus; Dara: daratumumab; DCd: daratumumab/cyclophosphamide; DRd: daratumumab/lenalidomide/dexamethasone; DVd, daratumumab/bortezomib/dexamethasone; GCV, ganciclovir; KPd, carfilzomib/pomalidomide/dexamethasone; Len, lenalidomide; mo, month; Rd, lenalidomide/dexamethasone; Ven, venetoclax; VGCV, valganciclovir; VRd, bortezomib/lenalidomide/dexamethasone; wk, weeks.

Abbreviations: IVT, intravitreal therapy; ND, not described; PD, progressive disease.

## Data Availability

Data are available on request due to privacy/ethical restrictions.
